# Evaluation of Acute Supplementation With the Ketone Ester (*R*)-3-Hydroxybutyl-(R)-3-Hydroxybutyrate (deltaG) in Healthy Volunteers by Cardiac and Skeletal Muscle ^31^P Magnetic Resonance Spectroscopy

**DOI:** 10.3389/fphys.2022.793987

**Published:** 2022-01-31

**Authors:** Donnie Cameron, Adrian Soto-Mota, David R. Willis, Jane Ellis, Nathan E. K. Procter, Richard Greenwood, Neil Saunders, Rolf F. Schulte, Vassilios S. Vassiliou, Damian J. Tyler, Albrecht Ingo Schmid, Christopher T. Rodgers, Paul N. Malcolm, Kieran Clarke, Michael P. Frenneaux, Ladislav Valkovič

**Affiliations:** ^1^Norwich Medical School, University of East Anglia, Norwich, United Kingdom; ^2^Department of Radiology, C.J. Gorter Center for High-Field MRI, Leiden University Medical Center, Leiden, Netherlands; ^3^Department of Physiology, Anatomy and Genetics, University of Oxford, Oxford, United Kingdom; ^4^Division of Cardiovascular Medicine, Radcliffe Department of Medicine, Oxford Centre for Clinical Magnetic Resonance Research, University of Oxford, Oxford, United Kingdom; ^5^Radiology Department, Norfolk and Norwich University Hospital, Norwich, United Kingdom; ^6^GE Healthcare, Munich, Germany; ^7^High Field MR Center, Center for Medical Physics and Biomedical Engineering, Medical University of Vienna, Vienna, Austria; ^8^Department of Clinical Neurosciences, Wolfson Brain Imaging Centre, University of Cambridge, Cambridge, United Kingdom; ^9^Department of Imaging Methods, Institute of Measurement Science, Slovak Academy of Sciences, Bratislava, Slovakia

**Keywords:** ketone monoester, ketone bodies, phosphorus MRS, ^31^P-MRS, heart, skeletal muscle, 3T, 7T

## Abstract

In this acute intervention study, we investigated the potential benefit of ketone supplementation in humans by studying cardiac phosphocreatine to adenosine-triphosphate ratios (PCr/ATP) and skeletal muscle PCr recovery using phosphorus magnetic resonance spectroscopy (^31^P-MRS) before and after ingestion of a ketone ester drink. We recruited 28 healthy individuals: 12 aged 23–70 years for cardiac ^31^P-MRS, and 16 aged 60–75 years for skeletal muscle ^31^P-MRS. Baseline and post-intervention resting cardiac and dynamic skeletal muscle ^31^P-MRS scans were performed in one visit, where 25 g of the ketone monoester, deltaG^®^, was administered after the baseline scan. Administration was timed so that post-intervention ^31^P-MRS would take place 30 min after deltaG^®^ ingestion. The deltaG^®^ ketone drink was well-tolerated by all participants. In participants who provided blood samples, post-intervention blood glucose, lactate and non-esterified fatty acid concentrations decreased significantly (−28.8%, *p* ≪ 0.001; −28.2%, *p* = 0.02; and −49.1%, *p* ≪ 0.001, respectively), while levels of the ketone body D-beta-hydroxybutyrate significantly increased from mean (standard deviation) 0.7 (0.3) to 4.0 (1.1) mmol/L after 30 min (*p* ≪ 0.001). There were no significant changes in cardiac PCr/ATP or skeletal muscle metabolic parameters between baseline and post-intervention. Acute ketone supplementation caused mild ketosis in blood, with drops in glucose, lactate, and free fatty acids; however, such changes were not associated with changes in ^31^P-MRS measures in the heart or in skeletal muscle. Future work may focus on the effect of longer-term ketone supplementation on tissue energetics in groups with compromised mitochondrial function.

## Introduction

Cardiac and skeletal muscle function declines with age, impairing quality of life in older individuals in a vicious cycle of decreased physical activity and muscle loss. Reduced myocardial function can promote physical inactivity, leading, in turn, to skeletal muscle dysfunction, and this becomes yet more pronounced in cardiac pathologies such as chronic heart failure. Even in relatively healthy older adults, the progressive loss of skeletal muscle mass and overall diminished strength with ageing can eventually develop into sarcopenia (Cruz-Jentoft et al., 2010), a condition estimated to affect up to 10% of the global population (Shafiee et al., 2017). The causes of this phenomenon are multi-factorial: hormonal changes with age, denervation of neuromuscular junctions, increased adiposity of skeletal muscle, and inflammatory infiltration, oxidation, or glycation of actin and myosin filaments may all contribute to the development of sarcopenia (Degens, 2007; Cruz-Jentoft et al., 2010; Walston, 2012; Marty et al., 2017; Shafiee et al., 2017). The emergence of overt mitochondrial dysfunction with ageing is also a distinct feature of cardiac and skeletal muscle dysregulation. Studies in mice have shown that declining skeletal muscle functional capacity with age is correlated with mitochondrial dysfunction, with uncoupled mitochondrial respiration, increased reactive oxygen species generation, and altered glucose homeostasis being more apparent in older animals (Jang et al., 2010; Lee et al., 2010). In older humans, loss of skeletal muscle mitochondrial function is correlated with diminished physical function (Joseph et al., 2012). Uncoupled mitochondrial respiration has also been documented in heart failure, (Neubauer, 2007) particularly when circulating free-fatty acid concentrations are high.

Ketone monoesters represent a promising substrate for addressing the energetic impairments of pathologies such as heart failure and sarcopenia, offering an exogenous, rapidly available alternative to fatty acids or carbohydrates. The importance of nutrition in ameliorating sarcopenia has been recognised (Welch et al., 2019), and the therapeutic benefits of ketogenic diets and nutritional ketosis have been examined in the context of this disease (Merra et al., 2016; Sammarco et al., 2017), but studies in sarcopenia to date have not assessed mitochondrial function. Furthermore, whereas typical dietary interventions take several days to produce useful levels of ketone bodies, similar blood concentrations can now be reached within 30 min through ingestion of the ketone monoester, (*R*)-3-hydroxybutyl (*R*)-3-hydroxybutyrate. These exogenous ketones have been shown to improve cardiac metabolic efficiency and endurance in rats (Sato et al., 1995; Murray et al., 2016), as well as the intramuscular energy balance during exercise in humans with a fatty acid oxidation disorder (Bleeker et al., 2020), as assessed by phosphorus magnetic resonance spectroscopy (^31^P-MRS). Indeed, ^31^P-MRS represents an ideal non-invasive modality for assessing the effects of pharmaceutical interventions on tissue bioenergetics *in vivo*. Primary metabolic parameters assessed by ^31^P-MRS in muscle tissue—namely, the cardiac phosphocreatine to adenosine triphosphate ratio (PCr/ATP) and the rate of recovery of intra-muscular PCr after exercise—have been linked to mitochondrial health (Fabbri et al., 2017; Zane et al., 2017). Further, similarly to cardiac PCr/ATP (Esterhammer et al., 2014), the skeletal muscle PCr recovery rate has also been shown to decline with age, with a marked impairment in pre-frail older adults (Andreux et al., 2018).

In this acute intervention study, we used ^31^P-MRS to investigate the potential benefit of nutritional ketone supplementation in both younger and older adults by studying changes in cardiac PCr/ATP and skeletal muscle PCr recovery following exercise, before and after ingestion of a ketone ester drink.

## Methods

### Study Population

For cardiac MRS, 12 healthy adults (6 male and 6 female), mean age (range) = 38 (23–70) years were recruited via advertisements displayed at the University of Oxford and on the John Radcliffe Hospital website. For skeletal muscle MRS, a total of 16 older adults (10 male and 6 female), mean age (range) = 67 (60–75) years were recruited through advertisements posted on public notice boards in the Norfolk community. Participants were asked to fast for 24 h prior to their visit, in order to increase free-fatty acids in blood, but they were encouraged to drink water. Exclusion criteria included: chronic clinical diseases such as cardiovascular disease, diabetes, renal impairment, neurological disorders, or diseases that may affect motor or cognitive function, except for hypertension and hyperlipidaemia; contraindications for undergoing magnetic resonance imaging and spectroscopy; and participation in ketogenic diets.

The cardiac protocol was preregistered at the ISRCTN (15716557) and approved by the Medical Sciences Interdivisional Research Ethics Committee of the University of Oxford (R50081/RE001). Skeletal muscle procedures were approved by the East of England – Cambridge Central Research Ethics Committee (18/EE/0111). Written informed consent was obtained in all cases and all study activities complied with the Declaration of Helsinki.

### Study Design

This study was designed as a single-arm clinical study where baseline and post-intervention ^31^P-MRS were performed in one visit. Subjects underwent either cardiac MRS or skeletal muscle MRS, but not both. After the baseline scan, each subject drank 25 mL of ketone ester, (*R*)-3-hydroxybutyl (*R*)-3-hydroxybutyrate (deltaG^®^, TΔS Limited, Thame, United Kingdom), timed so that the post-intervention ^31^P-MRS acquisition would take place 30 min after ingestion of deltaG^®^, when βHB concentrations were near maximal (Stubbs et al., 2017).

### Blood Metabolic Changes

Cardiac MRS participants gave their first blood sample immediately prior to drinking the ketone ester and undergoing the second scan. The second blood sample was obtained at the end of the second scan, approximately 40 min after participants drank the ketone monoester. Plasma from these blood samples was obtained by centrifugation and analysed for blood glucose, lactate, non-esterified fatty acids, and the ketone body D-beta-hydroxybutyrate using a commercial semi-automated bench-top analyser (ABX Pentra, Montpellier, France).

### Muscle Strength

Skeletal muscle MRS participants underwent muscle strength testing prior to MR procedures, to determine suitable ankle weights for exercise ^31^P-MRS. Maximum quadriceps muscle strength was defined as the highest of three consecutive values of torque (Nm) measured by left-leg knee extensor contraction at a knee flexion of 90° using a hand-held dynamometer (Lafayette Manual Muscle Testing System Model-01165, Lafayette Instrument Company, Lafayette, IN, United States). Torque was determined as the force generated during knee extension multiplied by the distance from the centre of the knee to the point where the dynamometer was applied to the tibia.

### Phosphorus Magnetic Resonance Spectroscopy

*Cardiac MR experiments.* Cardiac ^31^P-MRS scans were performed on a whole body 7 Tesla Magnetom MR scanner (Siemens Healthineers, Erlangen, Germany) following procedures described previously by Ellis et al. (2019). Subjects were scanned head-first supine. A 10 cm ^1^H transmit/receive loop coil (Rapid Biomedical, Rimpar, Germany) was used to acquire two-chamber, four-chamber and mid-short-axis spoiled gradient-recalled echo stacks of localiser cine images. The ^1^H coil was then replaced with a 16-element ^31^P array coil (Rapid Biomedical) consisting of a single rectangular 28 cm^2^ × 27 cm^2^ transmit loop and a 4 × 4 matrix of 16 circular flexible receive loops, each 5.5 cm in diameter. The ^31^P coil was placed in the same position as the ^1^H coil, above the interventricular septum. Serial, non-localised inversion-recovery acquisitions were performed for the post-hoc determination of transmit efficiency, using measurements from a central spherical phenylphosphonic acid (PPA) fiducial mounted on the coil housing. Custom MATLAB code was then used to determine the coil position from three orthogonal, single-channel ^31^P spoiled gradient-recalled echo images, localising five PPA fiducials including the centre fiducial used for B_1_ determination. B_0_ shimming was performed using a custom algorithm, described by Ellis et al. (2019). A short 3D ultra-short echo time chemical shift imaging (CSI) sequence was used for signal localisation, with the following parameters: repetition time = 1 s; field-of-view = 200 mm^3^ × 240 mm^3^ × 200 mm^3^; matrix size = 8 × 16 × 8; nominal voxel size = 9.4 mL; flip angle at interventricular septum = approximately 30°; shaped radiofrequency excitation pulse with bandwidth = 2,000 Hz; acquisition weighting with 4 averages at *k* = 0; and whitened singular value decomposition (WSVD) coil combination. The CSI grid was planned on a short-axis view of the heart, with the in-plane matrix parallel to the chest wall. The CSI matrix was fixed at the point of acquisition, and not shifted in post-processing. In order to minimise skeletal muscle signal contamination, a 25 mm thick, B_1_-insensitive train to obliterate signal (BISTRO) saturation band was placed in the anterior chest wall (Luo et al., 2001), The excitation pulse was centred at +266 Hz relative to PCr to cover metabolites from 2,3-diphosphoglycerate (2,3-DPG) to γ-ATP. Respiratory gating and ECG triggering were not used. The total time per exam, including participant set-up, cardiac imaging, and ^31^P-MRS, was approximately 30 min.

*Skeletal muscle MR experiments*. All skeletal muscle ^31^P-MRS was performed on a 3 tesla MR750w MRI scanner (GE Healthcare, Milwaukee, WI, United States) equipped with a 15 cm transmit-receive loop coil tuned to phosphorus (PulseTeq, Surrey, United Kingdom). Participants were positioned feet-first and supine with the loop coil fastened over their right vastus lateralis, using hook-and-loop straps, midway between the greater trochanter and lateral femoral epicondyle. Participants were then shifted laterally to place the right thigh close to the magnet’s isocentre, and additional hook-and-loop straps were applied to their knees to limit gross displacements during the scan. An inversion-recovery fast-spin-echo localiser was applied to verify that the cluster of three fiducials at the centre of the coil was positioned over the vastus lateralis. Then, after manual first-order B_0_ shimming, a non-localised ^31^P-MRS acquisition was performed with a Bloch-Siegert preparation pulse, provided as part of the MNS Research Pack (GE Healthcare, Munich, Germany), to determine the transmit gain needed to achieve a 90° excitation (Schulte et al., 2011). Exercise MRS was applied using a similar design to that described by Sleigh et al. (2016a). Briefly, the 12-min protocol consisted of two bouts, each comprising a 1-min rest period, a 1-min exercise period, and a 4-min post-exercise recovery period. The pulse-acquire ^31^P-MRS acquisition ran continuously over this 12-min protocol with the following sequence parameters: 240 dynamics with a repetition time = 3 s, spectral bandwidth = 2,250 Hz, 2,048 sampled points, a resonance frequency offset of 50 Hz, and block excitation with a flip angle of 90°. The total time per exam, including participant set-up, and skeletal muscle imaging and ^31^P-MRS, was approximately 40 min. Participants were given at least a 30-min break between baseline and post-intervention exams to minimise any effect of fatigue on post-intervention PCr recovery.

### Post-processing

*Cardiac muscle spectra.* Cardiac spectra from the midseptal voxel were fitted using the Oxford Spectroscopy Analysis (OXSA) toolbox—a MATLAB implementation of the AMARES fitting algorithm (Purvis et al., 2017). The fitted peaks, and their estimated chemical shifts, were: PCr at 0 ppm, γ-ATP at −2.48 ppm, and 2,3-diphosphoglyceric acid (2,3-DPG) at 5.4 and 6.4 ppm. The reported cardiac PCr/ATP values were calculated using the γ-ATP signal, corrected for blood signal contamination via subtraction of 15% of the total 2,3-DPG signal from the measured γ-ATP amplitudes (Rodgers et al., 2014), and corrected for partial saturation using relaxation times from the literature (Rodgers et al., 2014). Saturation factors were also adjusted for the actual flip angle in the interventricular septum, which was determined from coil position and transmit efficiency measures using the Biot-Savart law. Cramér-Rao lower bounds (CRLBs) were used to express the uncertainty in metabolite concentrations.

*Skeletal muscle spectra.* Skeletal muscle data were processed using an in-house software pipeline written in MATLAB (2018a, The Mathworks, Natick, MA, United States) and based on a previously-described routine for processing static ^31^P spectra (Cameron et al., 2019). Briefly, the pipeline comprised 15 Hz Lorentzian line-broadening, two-times zero-filling, zero- and first-order phase correction, frequency and phase alignment of individual dynamic spectra (Chen et al., 2002; Wiegers et al., 2017), and averaging of spectra in blocks of three. Each series of 80 spectra was then piped into the AMARES algorithm from jMRUI (version 3.0)^1^ (Vanhamme et al., 1997; Naressi et al., 2001; Stefan et al., 2009), where residual phase errors were corrected, and all metabolite signals were fitted and their amplitudes and chemical shifts calculated: PCr at 0 ppm, γ-ATP at −2.41 ppm, α-ATP at −7.51 ppm, β-ATP at −16 ppm, phosphodiesters (PDE) at 3 ppm, and inorganic phosphate (Pi) at 5.1 ppm. PCr breakdown during exercise was determined, and its recovery after exercise cessation was fitted using a mono-exponential rise to a maximum, as follows:


(1) $$\text{PCr}\,\left(t\right)={\text{PCr}}_{\text{e}}-\Delta \,\text{PCr}\cdot \exp^{-t\,/\,\tau_{\text{PCr}}}$$

where PCr_e_ is the post-recovery PCr signal, ΔPCr is the difference between the post-recovery and end-of-exercise PCr signals, and *τ*_PCr_ is the time constant of PCr resynthesis. Cytosolic pH was also estimated for each time-point of the dynamic ^31^P series using the Henderson-Hasselbalch equation:


(2) $$\text{pH}=6.75+\log\left(\frac{\delta -3.27}{5.63-\delta }\right)$$

where δ is the chemical shift between Pi and PCr. Acidosis was deemed to have occurred if the pH during exercise dropped by more than 0.2 units from the baseline value (Meyerspeer et al., 2020).

Further exercise MRS measures were calculated using the parameters derived above, including the initial PCr recovery rate:


(3) $${\text{V}}_{\text{PCr}}=\Delta \,\text{PCr}\,/\,\tau_{\text{PCr}}$$

the free adenosine diphosphate concentration:


(4) $$\left[\text{ADP}\right]=\left\{\left(\left[\text{TCr}\right]\,/\,\left[\text{PCr}\right]\right)-1\right\}\cdot \left[\text{ATP}\right]\,/\,\left(K\,\left[{\text{H}}^{+}\right]\right)$$

where the equilibrium constant *K* = 1.66 × 10^9^ L/mol, [H^+^] = 10^–pH^, and [ATP] and total creatine [TCr] = 8.2 and 42.5 mmol/L cellular water, respectively (Kemp et al., 2007); and the maximal rate of oxidative ATP synthesis:


(5) $${\text{Q}}_{\text{max}}=\left({\text{V}}_{\text{PCr}}+Q_{\text{B}}\right)\,\left\{1+{\left({\text{K}}_{\text{m}}\,/\,{\left[\text{ADP}\right]}_{\mathrm{end}\,\_\,\mathrm{exercise}}\right)}^{n}\right\}$$

where K_m_ is the [ADP] at the half-maximal oxidation rate, assumed to be 30 μM (Kemp and Radda, 1994), Q_b_ is the basal rate of ATP turnover, estimated at 0.04 mM/s (Jeneson et al., 2009), and *n* is the Hill coefficient.

Rest spectra were generated by averaging data from the initial one-minute rest period prior to exercise, for both baseline and post-intervention scans. Fitted peak intensities were relaxation-corrected using T_1_ values reported by Bogner et al. (2009), and metabolite concentrations were quantified, again assuming an ATP concentration of 8.2 mM/L cellular water (Kemp et al., 2007). Further, ratios of Pi to PCr and PCr to total phosphate were also calculated, where the latter represents the total area under all defined resonances in the spectrum: namely, Pi, PDE, PCr, and the three ATP resonances.

### Sample Size

Our preliminary work led to the hypothesis that ingestion of 25 g of the ketone ester (*R*)-3-hydroxybutyl (*R*)-3-hydroxybutyrate would lead to an increase of 22 and 15% in the primary outcome measures, cardiac PCr/ATP and skeletal muscle *τ*_PCr_, respectively. For cardiac experiments, the variance of PCr/ATP at 7 tesla was assumed to be 22%, based on work by Ellis et al. (2019). Thus, for a Type I error rate, α = 0.05, and a Type II error rate, β = 0.2—namely, 80% power—a sample size of *n* = 12 was required to reject the null hypothesis. Based on the results of Sleigh et al. (2016a), whose work formed the basis of our exercise ^31^P-MRS protocol, a variance of 10% or less was assumed for skeletal muscle *τ*_PCr_. Selecting a Type I error rate, α = 0.05, and a Type II error rate, β = 0.05—namely, 95% power—a sample size of *n* = 12 would be sufficient to reject the null hypothesis. To allow for drop-outs and technical issues, this recruitment target was increased to *n* = 16. Sample sizes were calculated using G^*^Power software (version 3.1, Heinrich-Heine-Universität Düsseldorf, Düsseldorf, Germany).

### Statistical Analysis

All statistical analyses were performed in R (Version 3, R Foundation for Statistical Computing, Vienna, Austria) and Microsoft Excel (Microsoft, Redmond, WA, United States). Data were tested for normality using the Shapiro-Wilk test. Differences between groups were assessed using two-sided Student’s *t*-tests when data were normally distributed and Mann-Whitney *U* tests when they were not, and correlations with age were explored using Pearson’s *r*. A *p*-value less than 0.05 was considered statistically significant in all analyses.

## Results

**FIGURE 1:**
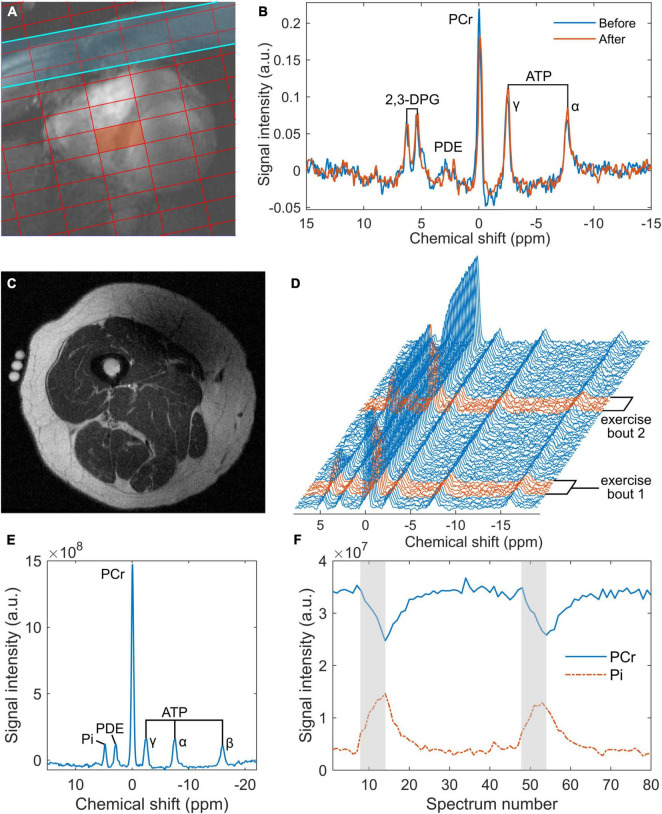
Representative data from the 7 tesla ^31^P cardiac magnetic resonance spectroscopy (MRS) acquisition **(A,B)** and the 3 tesla skeletal muscle acquisition **(C–F)**: **(A)** an example of localisation showing a chemical shift imaging grid superimposed on a short-axis, spoiled gradient-recalled echo cine image of the heart, where the red rectangle denotes the voxel of interest and the blue strip represents a saturation band used to suppress signal from skeletal muscle; **(B)** cardiac ^31^P spectra obtained from the septal voxel shown in a, pre- and post-intervention (‘Before’ and ‘After’, respectively); **(C)** an inversion recovery fast spin echo localiser image of a participant’s right thigh, showing fiducials at the centre of the 15 cm ^31^P coil positioned over the vastus lateralis; **(D)** a series of 80 ^31^P spectra averaged in groups of 3 from the 240 spectra acquired during rest and exercise—exercise spectra are highlighted in red; **(E)** an average rest spectrum obtained during the one-minute rest period at the beginning of the ^31^P-MRS acquisition; and **(F)** time courses of phosphocreatine (PCr) and inorganic phosphate (Pi) derived from peak fitting of the dynamic spectra from panel **(D)**, with exercise periods indicated in grey. ATP, adenosine triphosphate; a.u., arbitrary units; 2,3-DPG, 2,3-diphosphoglyceric acid; PCr, phosphocreatine; PDE, phosphodiesters.

For cardiac experiments, a total of 12 participants were enrolled, with no exclusions. Of the 16 participants recruited for skeletal muscle MRS, the ^31^P-MRS datasets of four were excluded due to hardware failure and a further two were discarded due to insufficient PCr breakdown. Participant demographics, after exclusions, are shown in Table 1. Ingestion of the deltaG^®^ ketone drink was well-tolerated by all participants. There were no reports of nausea or lower intestinal complaints, such as intestinal cramps or diarrhoea. Figure 1 shows representative data from the cardiac and skeletal muscle examinations.

**TABLE 1:** Participant characteristics.

	**Cardiac cohort**	**Skeletal muscle cohort**
Sex	6 male, 6 female	5 male, 5 female
Age, years	38.0 [23–70]	67.3 [60–75]
Height, cm	175 (9)	171 (13)
Weight, kg	69.8 (11.7)	74.1 (12.6)
BMI, kg/m^2^	22.9 (2.3)	24.8 (4.2)
Knee extension strength, N⋅m	–	134 (51.6)

*Data are expressed as mean (standard deviation) or mean [range].*

### Blood Metabolic Changes

**FIGURE 2:**
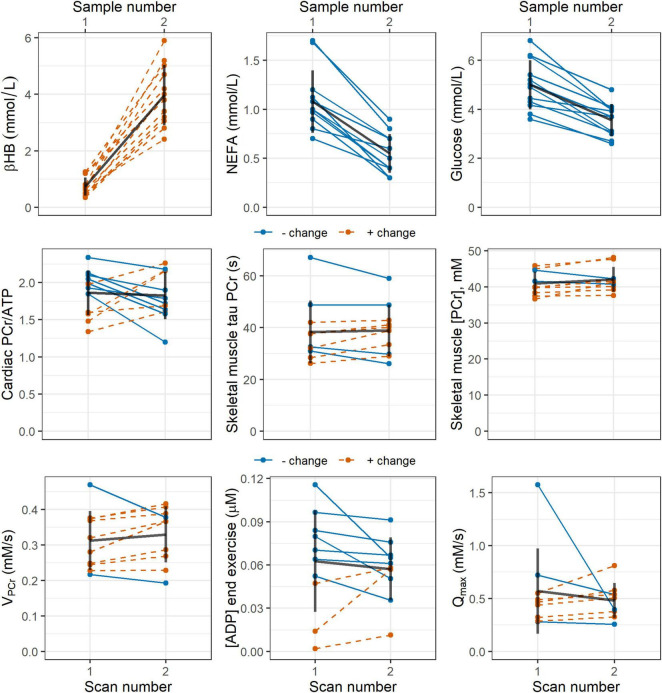
Line plots showing pre- and post-intervention changes in: (top row) blood concentrations of the ketone body D-beta-hydroxybutyrate (βHB), non-esterified fatty acids (NEFA), and glucose; and (middle and bottom rows) ^31^P magnetic resonance spectroscopy measures of cardiac PCr/ATP; and skeletal muscle tau PCr, PCr/ATP, ADP, initial PCr recovery rate V_PCr_, and maximal rate of oxidative ATP synthesis Q_max_. Blue, solid lines indicate participants who showed a negative change in the stated parameter, while red, dashed lines represent a positive change. Black lines show the mean change as well as the standard deviation pre- and post-intervention. ADP, adenosine diphosphate; ATP, adenosine triphosphate; PCr, phosphocreatine.

In the subset of participants who provided blood samples (*n* = 12), post-intervention blood glucose, lactate, and non-esterified fatty acid concentrations were significantly lower than pre-intervention values: mean (SD) = 5.00 (1.01) vs 3.56 (0.66) mmol/L, *p* ≪ 0.001; 1.84 (0.45) vs 1.32 (0.32), *p* = 0.02; and 1.08 (0.32) vs 0.55 (0.20) mmol/L, *p* ≪ 0.001, respectively, Blood levels of the ketone body D-beta-hydroxybutyrate were significantly higher after the intervention: mean (SD) = 0.73 (0.32) vs 3.98 (1.07) mmol/L, *p* ≪ 0.001. These changes are represented graphically by line plots shown in Figure 2 (top row).

### Cardiac Muscle Experiments

Cardiac ^31^P-MRS results are listed in Table 2 and shown graphically in Figure 2 (middle row). CRLBs for all spectra were less than 15% on average. There was no statistically-significant difference in cardiac PCr/ATP post-intervention relative to the pre-intervention value.

**TABLE 2:** Cardiac and skeletal muscle ^31^P-MRS parameters, pre- and post-intervention.

	**Pre-intervention**	**Post-intervention**	**Mean difference**	***p*-value**
**Cardiac ^31^P-MRS**				
PCr/ATP	1.86 (0.30)	1.83 (0.32)	−0.04 (0.42)	0.75
**Skeletal muscle ^31^P-MRS**				
Mean τ_PCr_, s	38.4 (12.2)	38.9 (10)	0.53 (4.63)	0.92
Mean PCr breakdown, %	32.5 (9.10)	34.4 (10.2)	1.90 (3.00)	0.67
V_PCr_ mM/s	0.31 (0.10)	0.33 (0.08)	0.02 (0.05)	0.29
[ADP] end exercise, μM	62.6 (35.2)	57.2 (21.9)	−5.3 (24.9)	0.52
Q_max_, mM/s	0.57 (0.40)	0.48 (0.40)	−0.09 (0.42)	0.55

Resting [PCr], mM	40.9 (3.26)	42.1 (3.41)	1.18 (2.04)	0.44
Resting [Pi], mM	4.64 (1.18)	4.75 (1.20)	0.11 (1.07)	0.84
Resting [PDE], mM	7.04 (1.40)	7.04 (1.27)	0 (0.88)	0.99
Resting Pi/PCr	0.12 (0.02)	0.13 (0.01)	0 (0.02)	0.59
Resting PCr/Total-phosphate	0.53 (0.02)	0.54 (0.02)	0.01 (0.01)	0.5
Resting pH	7.19 (0.24)	7.21 (0.15)	0.02 (0.13)	0.82

*The *p*-values listed were obtained from two-sided Student’s *t*-tests. Data are represented as mean (SD).*

*ATP, adenosine triphosphate; PCr, phosphocreatine; PDE, phosphodiesters; Pi, inorganic phosphate; Q_*max*_, maximal rate of oxidative ATP synthesis; τ_*PCr*_, time constant of PCr resynthesis; V_*PCr*_, initial rate of PCr resynthesis.*

*Cardiac phosphorus MRS parameters versus age.* Relationships between cardiac PCr/ATP and age were explored using Pearson’s *r*. There was no correlation between PCr/ATP and age at baseline (*r* = −0.43, *p* = 0.16) and neither was there a correlation between change in PCr/ATP and age (*r* = 0.21, *p* = 0.52).

### Skeletal Muscle Experiments

All ^31^P-MRS-derived parameters for skeletal muscle are shown in Table 2, and selected parameters are illustrated in line plots in Figure 2 (middle and bottom rows). There were no statistically significant differences in the means of any ^31^P-MRS parameters post-intervention relative to pre-intervention. No datasets appeared to show acidosis, either pre- or post-intervention.

*Skeletal muscle phosphorus MRS parameters versus age.* There were no statistically-significant correlations between baseline tau PCr and age or change in tau PCr and age shown by Pearson’s *r* (*r* = 0.03, p = 0.93; and *r* = 0.47, *p* = 0.17, respectively). There were also no correlations observed between: baseline [PCr] and age or change in [PCr] and age (*r* = −0.31, *p* = 0.38; and *r* = −0.18, *p* = 0.62, respectively); baseline V_PCr_ or change in V_PCr_ and age (*r* = −0.10, *p* = 0.79; and r = −0.34, *p* = 0.33); or baseline Q_max_ or change in Q_max_ and age (*r* = 0.41, *p* = 0.28; and r = −0.36, *p* = 0.34).

## Discussion

In this study we conducted an acute intervention of a single ketone ester drink in a cohort of healthy adults, where cardiac and skeletal muscle high-energy phosphate metabolism was monitored via ^31^P-MRS immediately before, and 30 min after, ketone administration. Post-intervention, we saw significantly increased blood concentrations of the ketone body D-beta-hydroxybutyrate and a significant decrease in concentrations of glucose, lactate, and non-esterified fatty acids. However, we did not observe any statistically-significant post-intervention changes in cardiac PCr/ATP ratios, or in skeletal muscle PCr recovery rates or metabolite ratios.

One of the primary strengths of this study was the use of ^31^P-MRS, which is a sensitive and widely-used method for assessing cardiac and skeletal muscle energetics. We used established protocols for both cardiac and skeletal muscle ^31^P-MRS, taking particular care with regards to repeatability aspects. For cardiac ^31^P-MRS, we elected to avoid cardiac triggering and respiratory gating to maintain consistent repetition times and avoid unpredictable extensions to scan time. Our assessment of cardiac PCr/ATP was done based on the mid-septal voxel signal, which has been shown to give the most reproducible results (Ellis et al., 2019). For the skeletal muscle ^31^P-MRS protocol, we averaged the results of two exercise bouts together to improve the precision of our exercise MRS measures, in line with the findings of Sleigh et al. (2016a). Thus, taking these considerations into account, it is unlikely that the absence of significant differences in ^31^P-MRS parameters pre- and post-intervention was due to a lack of measurement power (Ellis et al., 2019).

The results we show here may indicate that the previously observed haemodynamic benefits of ketone supplementation (Sato et al., 1995; Nielsen et al., 2019) are not necessarily caused by an increased availability of ATP, but rather by improved efficiency of its utilisation. Veech has theorised that ketone bodies have a more electronegative character than other metabolites, having more hydrogen atoms per carbon (Veech, 2004). This could allow them to produce a larger proton gradient and, therefore, more efficient utilisation of ATP. This hypothesis could potentially be investigated *in vivo* using saturation transfer experiments at rest and during exercise. Such experiments can assess changes in Pi-to-ATP exchange rates due to increased workload (Sleigh et al., 2016b; Tušek Jelenc et al., 2016). Further exercise ^31^P-MRS analyses could also be performed to calculate the ΔG of ATP hydrolysis (Fiedler et al., 2016). Regardless, the fact that we show no post-intervention differences in ^31^P-MRS measures should not be interpreted as indicating a lack of potential benefits of long-term supplementation, or a lack of efficacy, because our participants were healthy volunteers and supplementation in heart failure patients has already proven to be beneficial (Nielsen et al., 2019). These considerations are also supported by a 1.5 tesla ^31^P-MRS study by Bleeker et al., who showed an increase in skeletal muscle Pi/PCr in patients deficient in very-long-chain acyl-CoA dehydrogenase after acute ketone supplementation (Bleeker et al., 2020), and a similar result was also seen in canine myocardium in an earlier study by Kim et al. (1991). As in this work, Bleeker and colleagues did not show changes in post-exercise PCr recovery after the ketone intervention. In contrast to the study design of Bleeker et al., participants in our study were asked to fast for 24 h prior to their visit to maximise blood levels of D-beta-hydroxybutyrate by reducing competition from other energy substrates in meals (Stubbs et al., 2017). In terms of the timing of our ^31^P-MRS procedures relative to ingestion of the deltaG^®^ drink, both we and Bleeker et al. administered the intervention 30 min prior to the scan (Bleeker et al., 2020). This timing was based on work by Stubbs et al. (2017), who showed that blood levels of D-beta-hydroxybutyrate reach a maximum between 30 and 60 min after administration—an interval that coincides with the data acquisition in our study. Nielsen et al. (2019) used a 3-h infusion of βHB salt as their intervention; however, this is not directly comparable to the approach we show here, as our deltaG^®^ drink is a food and, therefore, cannot be infused.

Based on previous data, we anticipated increases in the primary cardiac and skeletal muscle outcome measures, PCr/ATP and *τ*_PCr_, respectively, as a result of our acute ketone ester intervention. The rationale for this was based on the fact that fatty acids are the natural ligand of peroxisome proliferator-activated receptor alpha (PPARα), which upregulates the transcription of uncoupling protein 3 (UCP3), which, in turn, uncouples the mitochondrial membrane and reduces ATP production in the myocardium (Villarroya et al., 2007). Notably PCr/ATP correlates with free-fatty acid blood concentration (Scheuermann-Freestone et al., 2003), and plasma free-fatty acid concentrations correlate with uncoupling protein expression in the myocardium (Murray et al., 2004). Given the electronegative advantage of ketone bodies (Veech, 2004), we hypothesised that acute ketosis could improve free-fatty-acid-induced uncoupling and increase ATP concentrations, which could be detected with ^31^P-MRS. However, we were unable to show the expected positive results in ^31^P-MRS measures, despite the ketosis in blood in the studied participants. Since our study was powered to detect the anticipated positive effects—a 22% increase in cardiac PCr/ATP and a 15% increase in skeletal muscle *τ*_PCr_—we cannot rule out much smaller positive, or negative, effects. Our findings should serve as guidance in terms of possible effect sizes, while our study design and methodology will form a useful template for future investigations. Indeed, it is also worth noting here that the synthetic ketone ester we used was well-tolerated in the acute setting, in line with findings from a recent safety study on long-term ketone supplementation (Soto-Mota et al., 2019). This supports longer-term supplementation in future studies, which could also be monitored via ^31^P-MRS.

There are some limitations to the work reported here. We calculated metabolite concentrations in skeletal muscle based on an assumed ATP concentration of 8.2 mM, whereas ATP concentrations possibly decrease with age. However, we expect this change to be relatively consistent in our group of older participants, and intra-individual comparisons, pre- versus post-intervention, are not affected. Indeed, our theory that ketone supplementation leads to increased efficiency of ATP utilisation, and not increased ATP availability, lends itself to this assumption. Another possible limitation is that participants were not selected based on their level of physical fitness. Deconditioned individuals are expected to benefit more from ketone supplementation due to energetic deficits, manifesting as longer PCr recovery time constants and lower cardiac and skeletal muscle PCr/ATPs. Future studies could target older participants who demonstrate some of the hallmarks of frailty, as assessed by grip strength, walking speed, and physical performance batteries.

In conclusion, acute supplementation with a ketone ester drink in healthy volunteers caused mild ketosis in blood, with a concomitant drop in glucose, lactate, and free fatty acids; however, we were not able to detect an effect on ultra-high-field ^31^P-MRS measures in the heart, or in high-field MRS in resting and exercising skeletal muscle. Future work should focus on the effect of longer-term ketone supplementation on cardiac and muscle energetics, particularly in groups with compromised mitochondrial function.

## Data Availability Statement

The raw data supporting the conclusions of this article will be made available by the authors, without undue reservation.

## Ethics Statement

The studies involving human participants were reviewed and approved by the Medical Sciences Interdivisional Research Ethics Committee of the University of Oxford and the East of England - Cambridge Central Research Ethics Committee. The participants provided their written informed consent to participate in this study.

## Author Contributions

DC, AS-M, JE, NP, AS, CR, MF, KC, and LV: study conception and design. DC, AS-M, DW, JE, RG, and NS: acquisition of data. DC, AS-M, JE, and CR: analysis and interpretation of data. DC, AS-M, JE, NP, KC, and LV: drafting of manuscript. All authors: critical revision.

## Author Disclaimer

The views expressed are those of the author(s) and not necessarily those of the NIHR or the Department of Health and Social Care.

## Conflict of Interest

RS is employed by GE Healthcare. The intellectual property covering the uses of ketones and ketone esters is owned by BTG Ltd., the University of Oxford, the National Institutes of Health, and TΔS Ltd. Should royalties ever accrue from these patents, KC, as an inventor, will receive a share of the royalties under the terms proscribed by Oxford University. KC is a director of TΔS Ltd., a company spun out of the University of Oxford to develop and commercialise products based on the science of ketone bodies in human nutrition. The remaining authors declare that the research was conducted in the absence of any commercial or financial relationships that could be construed as a potential conflict of interest.

## Publisher’s Note

All claims expressed in this article are solely those of the authors and do not necessarily represent those of their affiliated organizations, or those of the publisher, the editors and the reviewers. Any product that may be evaluated in this article, or claim that may be made by its manufacturer, is not guaranteed or endorsed by the publisher.

## Footnotes

1
https://web.archive.org/web/20211011011850/http://jmrui.eu/
